# Air pollution, greenspace, and metabolic syndrome in older Czech and Swiss populations

**DOI:** 10.1097/EE9.0000000000000393

**Published:** 2025-05-08

**Authors:** Andrea Dalecká, Ayoung Jeong, Daniel Szabó, Balint Tamasi, Medea Imboden, Emmanuel Schaffner, Dirk Keidel, Youchen Shen, Mark Nieuwenhuijsen, Marta Cirach, Kees de Hoogh, Jelle Vlaanderen, Roel Vermeulen, Annette Peters, Erik Melén, Anne Peasey, Martin Bobák, Hynek Pikhart, Nicole Probst-Hensch

**Affiliations:** a RECETOX, Faculty of Science, Masaryk University, Brno, Czech Republic; b Swiss Tropical and Public Health Institute, Allschwil, Switzerland; c University of Basel, Basel, Switzerland; d Institute for Risk Assessment Sciences, Utrecht University, Utrecht, the Netherlands; e ISGlobal, Barcelona, Spain; f Institute of Epidemiology, Helmholtz Zentrum München - German Research Center for Environmental Health, Neuherberg, Germany; g IBE, Medical Faculty, Ludwig-Maximilians-Universität München, Munich, Germany; h German Center for Diabetes Research (DZD e.V.), Neuherberg, Germany; i Department of Clinical Science and Education, Södersjukhuset, Karolinska Institute, Stockholm, Sweden; j Institute of Epidemiology & Health Care, University College London, London, United Kingdom

**Keywords:** Air pollution, Cross-sectional design, Greenspace, Metabolic syndrome, Particulate matter

## Abstract

**Background:**

The prevalence of metabolic syndrome (MetS) has increased rapidly, with considerable variation between European countries. The study examined the relationship between air pollutants, greenspace, and MetS and its components in the Czech and Swiss populations.

**Methods:**

Cross-sectional data from the Czech Health, Alcohol and Psychosocial Factors in Eastern Europe (HAPIEE) (n = 4,931) and the Swiss cohort Study on Air Pollution and Lung and Heart Diseases in Adults (SAPALDIA) (n = 4,422) cohorts included participants aged 44–73 years. MetS was defined as abdominal obesity plus two additional components (hypertension, diabetes, low high-density lipoprotein cholesterol, and elevated triglycerides). Annual mean concentrations of PM_10_, PM_2.5_, NO_2_, and greenspace (defined as the annual mean of normalized difference vegetation index within 500 m) were assigned to the individual residential level. We estimated odds ratios (OR) using multivariable logistic regressions with cluster-robust standard error, controlling for multiple confounders.

**Results:**

The prevalence of MetS was significantly higher in the Czech (51.1%) compared with Swiss (35.8%) population as were the concentration means of PM_10_ and PM_2.5_. In HAPIEE, a 5 μg/m^3^ increase in PM_2.5_ was associated with 14% higher odds of MetS (OR = 1.14; 95% confidence interval [CI] = 1.01, 1.28). In SAPALDIA, no evidence was found for the associations between air pollutants and MetS (e.g. OR = 1.01; 95% CI = 0.90, 1.13 for PM_2.5_). No protective effects of normalized difference vegetation index on MetS were observed. Upon inspection of MetS components, PM_2.5_ and PM_10_ exposures were associated with higher odds of hypertension and elevated triglycerides in HAPIEE only, while PM_2.5_, PM_10,_ and NO_2_ were associated with higher odds of diabetes in SAPALDIA only.

**Conclusion:**

Individuals with higher exposures to PM_2.5_ may be at higher risk of MetS. The differential associations with MetS components between the cohorts deserve further investigation.

**What this study adds**

To the best of our knowledge, this is the first comparative analysis of Western and Eastern European countries providing evidence of relationships between environmental characteristics, including air pollution and greenness, and prevalence of metabolic syndrome and its components in two environmentally distinguished regions. The exposure estimation was performed using harmonized methods and unified metabolic syndrome definition improving comparability of the findings. The study confirms the value of including cohorts from different economic, environmental, behavioral, and health systems contexts for adapting environmental impact assessment.

## Introduction

Metabolic syndrome (MetS) is a spectrum of pathophysiological conditions accelerating the progression of cardiovascular disease.^1^ It is typically defined as an occurrence of a minimum of three of five components, including abdominal obesity, elevated blood pressure, diabetes, elevated triglycerides (TG), and low levels of high-density lipoprotein cholesterol (HDL).^2^ According to the Global Burden of Diseases, Injuries, and Risk Factors Study 2019, the prevalence rates of all metabolic components have risen globally, posing a major health and social burden in both low- and middle-income and high-income countries.^3^ Regional comparisons have generally reported a higher prevalence of MetS and its components in Eastern compared with Western European countries.^4,5^ Age, genetic predisposition, low physical activity, and unhealthy diet have been well-documented as crucial risk factors for MetS.^6–8^ In contrast, comparative evidence on long-term exposure to environmental factors, including air pollution and greenspace, remains inconclusive.^9^

Air pollution belongs among the top environmental risk for disease burden and have been previously associated with cardiovascular diseases and MetS.^9,10^ Systematic review and meta-analyses, including results from nine observational studies reported no significant associations between particulate matter with a diameter of 1 μm or less (PM_1_), particulate matter with a diameter of 2.5 μm or less (PM_2.5_), particulate matter with a diameter of 10 μm or less (PM_10_), or NO_2_ and occurrence of MetS.^9^ In contrast, findings from a Chinese nationwide study suggested statistically significant increased odds of MetS when exposed to PM_2.5_, PM_10_, SO_2_, NO_2,_ or ozone.^11^ Despite the inconsistency of the epidemiological findings on the association between air pollution and MetS, pathophysiological pathways potentially explaining the effect of ambient air pollutants on MetS have been recognized in human studies, including systemic inflammation and altered metabolism of lipids and glucose.^11–13^

Greenness exposure has been proposed to have beneficial effects on human health, including MetS. A recent systematic review and meta-analysis showed that an increase in the normalized difference vegetation index (NDVI) within a 500-m buffer was linked to lower odds of MetS.^14^ There are various mechanisms potentially underlying the protective effect of greenness on MetS, including the promotion of physical activity and social engagement; reduced stress; and mitigation of exposure to environmental exposures.^15,16^

To the best of our current knowledge, previous studies focusing on air pollution, greenness, and MetS were mainly conducted in Western Europe,^9,16–19^ the United States,^12,20^ and China.^11,21,22^ There is limited evidence from Eastern European areas, and no study so far provided a comparative analysis on both pollution domains between populations living in these regions. The comparative analyses between Western and Eastern European countries allow for a comprehensive understanding of how environmental factors influence MetS across varying contexts of air quality and greenspace, socioeconomic disparities, and public health policies. Within the EXposome Powered tools for healthy living in urbAN Settings (EXPANSE) project,^23^ we aimed to examine the associations of three main air pollutants (NO_2_, PM_10_, and PM_2.5_), and greenness with prevalence of MetS and its components using harmonized exposure models assigned to residential addresses in two well-established longitudinal cohort studies of comparable design established in the Czech Republic and Switzerland.

## Materials and methods

### Study populations

The study utilized data from (1) the Czech cohort arm of the Health, Alcohol and Psychosocial Factors in Eastern Europe (HAPIEE),^24^ and (2) the Swiss cohort Study on Air Pollution and Lung and Heart Diseases in Adults (SAPALDIA).^25,26^

**Figure 1.**
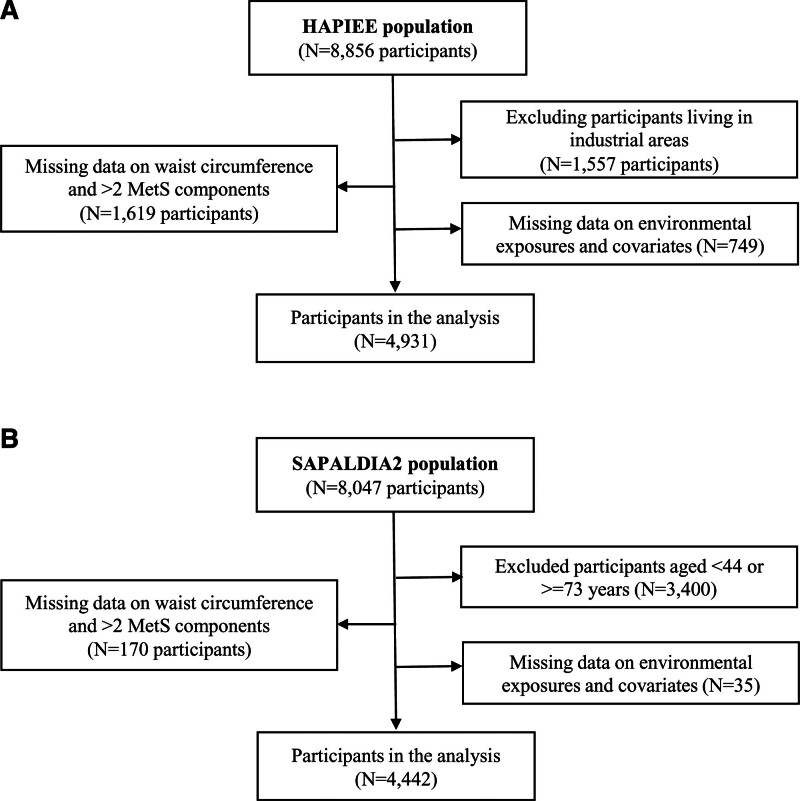
Flow charts of the HAPIEE (A) and SAPALDIA (B) cohorts. HAPIEE, Health, Alcohol and Psychosocial Factors in Eastern Europe; SAPALDIA, Swiss cohort Study on Air Pollution and Lung and Heart Diseases in Adults.

The HAPIEE cohort was established in 2002–2005 to investigate the impact of socioeconomic and psychosocial conditions on noncommunicable diseases in four postcommunist Central and Eastern European countries (Czech Republic, Poland, Lithuania, and Russia).^24^ Only the Czech arm of the study was included due to the availability of the geocoded residential addresses needed for estimating environmental exposures. The Czech HAPIEE cohort enrolled participants from seven urban areas and consisted of 8856 subjects at baseline. For this study, we excluded participants living in two highly industrial areas to control for potential occupational bias (Figure 1A).

The SAPALDIA cohort, involving eight geographical areas of Switzerland, was initiated in 1991 to examine the associations between long-term exposure to air pollution and respiratory, cardiovascular, and metabolic health. As previously described in detail,^26^ 9651 participants were examined in 1991 (SAPALDIA1), and 8047 (86% of alive persons) out of the 9651 participants were followed up in SAPALDIA2 (Figure 1B).

The analytical samples involved participants from the baseline investigation of HAPIEE and the first follow-up of SAPALDIA to ensure that the data covered comparable age distributions and similar collection time points (2002–2005 for HAPIEE and 2000–2003 for SAPALDIA2). The current analysis ultimately consisted of men and women aged 44–73 years who underwent physical examinations, provided blood samples, and completed the relevant questionnaires at the relevant cohort time point.

The HAPIEE study was approved by the institutional ethics committees in all participating centers and the University College London. The SAPALDIA study was approved by the ethics committees of the participating cantons and the Swiss Academy of Medical Sciences. All participants provided written informed consent before participating in the studies (S9 information; https://links.lww.com/EE/A343).

### Outcome assessment: metabolic syndrome definition

The definition of MetS was applied according to the International Diabetes Federation Worldwide Definition using the following five components: abdominal (central) obesity (defined as waist circumference ≥94 cm in males and ≥80 cm in females); hypertension (systolic blood pressure ≥130 mm Hg or diastolic blood pressure ≥85 mm Hg and/or self-reported diagnosis and/or treatment for previously diagnosed hypertension); diabetes (fasting plasma glucose ≥5.6 mmol/L and/or self-reported diagnosis and/or treatment for previously diagnosed type 2 diabetes; fasting time was considered as at least 4 hours before the examination.); elevated TG (TG ≥1.7 mmol/L and/or specific treatment for this lipid abnormality); and low HDL cholesterol (<1.03 mmol/L in males and <1.29 mmol/L in females and/or treatment for lipid abnormality).^27^ According to the International Diabetes Federation definition, MetS was defined as the presence of abdominal obesity and any of the two above-listed criteria.

Blood samples were collected for laboratory analysis. In both studies, different venous blood fractions were collected and stored at −80 °C in the cohort’s biobanks. Information on the time since the last intake of calories was obtained to assess the fasting state. In SAPALDIA, A Hitachi Modular Autoanalyser (Rotkreuz, Switzerland) and assays from Roche Diagnostics (Mannheim, Germany) have been used to measure serum levels of TG and total cholesterol (all enzymatic tests). HDL was measured with a homogenous assay (Roche Diagnostics, Mannheim, Germany) using Roche Cobas Integra (Rotkreuz, Switzerland). HDL values were only used if the participants had a triglyceride level of 9.4 mmol/L or lower. Plasma glucose was measured from venous blood in all SAPALDIA participants.^28^ In HAPPIE, HDL-cholesterol and TG were measured on a Roche Cobas MIRA auto-analyzer with the use of a conventional enzymatic method with reagents from Boehringer-Mannheim Diagnostics and Hoffmann-La Roche.^29^ Plasma glucose was measured in venous blood in a subsample (N = 831) and in finger prick capillary blood using a Glucotrend glucometer.^29^ Previous studies suggested a strong correlation between venous and capillary blood glucose; however, our capillary samples showed a small but significant overestimation (by 10–15%), particularly in lower levels of blood glucose.^30,31^ Therefore, we conducted an imputation of missing values in venous blood glucose. First, we calculated the differences between venous glucose (measured in subsample) and capillary glucose (measured in whole sample). The differences within study sample were not consistent across the glucose levels due to increasing overestimation caused by glucometer measurements. Therefore, we calculated means and standard deviations separately for multiple glucose levels to better reflect on original distribution of differences observed between venous and capillary blood glucose. Subsequently, we generated random values with multiple repetitions representing glucose mean differences. Finally, we predicted venous glucose by using the prediction model as follows:


$$\begin{array}{l} Venous\;\;\;glucose\;\;\;\left(\frac{mmol}{L}\right)=\;\;\;\beta_{0}+\;\;\;\beta_{1}\;\;\;*\;\;\;capillary\;\;\;glucose\;\;\;\;+\;\;\;glucose\;\;\;mean\;\;\;difference \end{array}$$

The Pearson correlation between the predicted glucose and the actual glucose measured at subsample was r = 0.978.

Blood pressure was measured at rest, in a sitting position three times and twice, respectively, in HAPIEE and SAPALDIA. The mean values of measures were computed for analyses.

Waist circumference was measured in HAPIEE participants. In SAPALDIA, it was not measured at baseline and at the first follow-up, but only at the second follow-up about 10 years later (SAPALDIA3). Therefore, a prediction model was built to derive the waist circumference from the 2nd follow-up investigation conducted in 2010. The model is described elsewhere.^19^ Briefly, the prediction model, with optimal Bayesian Information Criterion, included various covariates such as sex, age, body mass index (BMI), alcohol, physical activity, and smoking status. The waist circumference for the present study was predicted from the covariate values from the second wave and residuals from the same model of the third wave, to back predict waist circumference for the present analyses. Additionally, the cross-validation was applied to assess the imputation model by randomly splitting the sample into a training and a validation sample.^19^

The MetS and its individual components were treated as binary outcomes in this analysis.

### Exposure assessment

The uniform exposure assessment developed within the EXPANSE project was followed for both cohorts. The EXPANSE project aims to disentangle the complex associations between the urban exposome on cardio-metabolic and pulmonary disease across Europe.^23^ The exposures were assigned to the residential addresses collected at baseline in HAPIEE and at the first follow-up in SAPALDIA, and thus to residential addresses valid at the time point of assessing the presence or absence of MetS.

#### Ambient air pollution

A land-use regression model was built using measured concentrations from routine European monitoring stations.^32,33^ Potential predictors included satellite retrievals, chemical transport model estimates, and land-use variables. Supervised linear regression was first used to select predictors, and then geographically weighted regression estimated the spatially and annually varying coefficients. Model performance was evaluated using five-fold cross-validation. A more detailed description of the air pollution model can be found elsewhere.^32^ For this study, we calculated weighted annual mean concentrations of PM_10_, PM_2.5_, and NO_2_ exposures from the annual average in the calendar year of the examination and the year before. The exposures of the air pollutants from 2000 to 2005 were utilized to cover the time frame of the examination periods of the HAPIEE and SAPALDIA2. The air pollution exposures were assigned to the participant’s home addresses on 100 × 100-m resolution.

#### Greenspace

Exposure to greenspace was characterized using the annual mean values of the NDVI within 500 m of the residential address estimated in 2000 to assess average surrounding greenness. NDVI quantifies photosynthetically active vegetation by measuring the difference between near-infrared and red light and ranges from +1.0 to −1.0.^34^ Values <0 were ignored as they represent water bodies.

### Covariates

The covariates included in a model were selected based on a previous literature review of the potential predictors of MetS.^35–37^ Age was coded as a continuous variable, while sex was assessed as a binary variable (men and women). Educational attainment was grouped into three categories, including “incomplete primary or primary education,” “vocational or secondary,” and “university or higher.” Marital status was classified into four categories, including “married/cohabiting,” “divorced/separated,” “widowed,” and “single.” Smoking status was defined by three categories, including “never,” “former,” and “current/occasional.” Self-reported physical activity was grouped into two categories of being “inactive,” and “active” that represented 0 hours/week, respectively >0 hours/week of performed vigorous activity. Frequency of alcohol consumption was defined by two groups, including “< once per month” and “≥ once per month.” Fruit and vegetable consumption was defined by two categories separately for the HAPIEE and SAPALDIA cohorts, reflecting meeting or not meeting World Health Organization (WHO) recommendations.^38^ In HAPIEE, meeting WHO recommendation was defined as consumption of at least 400 g of fruits and 400 g of vegetables per day. In SAPALDIA, meeting WHO recommendation was defined as the consumption of fruits and vegetables every day. To control for seasonal variation in performed physical activity, consumed fruit and vegetable, and environmental exposures, we additionally included the month of the year as a potential confounder. Finally, we considered the neighborhood-level socioeconomic position (nSEP) of participants derived from a principal component analysis separately in each cohort using various characteristics. In HAPIEE, nSEP was constructed from percent of unemployed population, percent of less than secondary educated population, and percent of university educated population. In SAPALDIA, nSEP was constructed from median rent, percent of households headed by a person with primary education or less, percent of households headed by manual or unskilled workers, and mean number of persons per room. A higher nSEP index indicated a higher socioeconomic position in both cohorts.^39^

### Statistical analyses

The descriptive characteristics of the cohort samples were assessed and presented separately. Categorical variables were described using frequencies, while continuous variables using means and standard deviations. We computed the Spearman correlation of environmental exposures and Tetrachoric correlation of MetS components in each cohort. Statistical approaches were selected according to the research questions. We conducted multivariable logistic regression to examine the individual effects of ambient air pollution exposures and greenspace on MetS and its components separately for each cohort. All models were adjusted for multiple confounders, including age, sex, education, marital status, smoking status, physical activity, alcohol consumption, fruit and vegetable consumption, month of the year, and nSEP. We applied cluster-robust standard errors to control for clustering respondents within 5 and 8 study areas in HAPIEE and SAPALDIA, respectively.^40^

The results are presented separately for each cohort and reported odds ratios (OR) together with corresponding 95% confidence intervals (CI) that represented a fold increase in odds of outcomes per 5 μg/m^3^ of PM_10_, PM_2.5_, NO_2_, and 0.1 units of NDVI. Data analyses were conducted using STATA software (version 16.0, StataCorp, College Station, TX).

### Sensitivity analysis

Given the relatively high prevalence of the outcome, we additionally estimated incidence rate ratios (IRR) for MetS using Poisson regression with cluster-robust standard errors. Finally, we investigated the associations between environmental exposures and MetS components in participants neither previously diagnosed with MetS nor treated for any of the components (N = 3066 for SAPALDIA and N = 2398 for HAPIEE). The MetS components were defined according to the measured biomarkers only.

## Results

### Description of study population

The analytical samples consisted of 4931 participants (54.8% females) and 4442 participants (51.8% females) from the HAPIEE and the SAPALDIA cohorts, respectively (Table 1). The average age of the participants was 58.19 years in HAPIEE and 57.53 years in SAPALDIA. In both cohorts, most individuals attained secondary education and were married or cohabited. There were comparable proportions of current (including occasional) smokers in the cohorts. Compared with SAPALDIA, HAPIEE participants were more physically active (57.8% in Swiss compared with 71.6% in Czech individuals) and consumed alcohol less frequently (Table 1). Czech individuals were substantially more exposed to air pollutants compared with Swiss participants, notably to particulate matter air pollution. The exposure to greenness in 500 m buffer was 0.54 and 0.45 in SAPALDIA and HAPIEE, respectively (Table 1). In HAPIEE, we observed a strong positive correlation between PM_10_ and PM_2.5_ (*P* < 0.001), while there was no correlation between particulate matters and NO_2_ (Table S1; https://links.lww.com/EE/A343). Correlations between all air pollutants were high in SAPALDIA (*P* < 0.001, Table S2; https://links.lww.com/EE/A343). Inverse correlations between air pollution, in particular NO_2_, with NDVI were higher in SAPALDIA than in HAPIEE, and correlations of all air pollutants with neighborhood SEP were positive in both cohorts.

**Table 1.** Descriptive characteristics of the analytical samples

	HAPIEE	SAPALDIA
	N = 4931	N = 4442
Age, mean (SD)	58.19 (7.15)	57.53 (7.77)
Gender, %		
Females	54.8	51.8
Males	45.2	48.2
Education, %		
Incomplete primary/primary	9.9	7.9
Vocational/secondary	74.4	66.9
University or higher	15.7	25.2
Marital status, %		
Married/cohabiting	75.8	73.6
Divorced/separated	12.6	10.2
Widowed	8.9	5.7
Single	2.7	10.5
Smoking status, %		
Never	45.2	40.8
Former	30.1	35.9
Current/occasional	24.7	23.3
Physical activity, %		
Inactive (0 h/w of vigorous activity)	28.4	42.2
Active (>0 h/w of vigorous activity)	71.6	57.8
Alcohol frequency, %		
<Once per month	36.8	28.6
≥Once per month	63.2	71.4
Fruit and vegetable consumption, %		
Not meeting WHO recommendation^a^	36.9	56.8
Meeting WHO recommendation	63.1	43.2
Air pollutants [μg/m^3^], mean (SD)		
PM_10_	35.94 (4.08)	23.04 (5.06)
PM_2.5_	26.62 (3.91)	16.20 (4.49)
NO_2_	26.47 (4.44)	25.68 (7.58)
Greenness, mean (SD)		
NDVI, 500 m	0.45 (0.07)	0.54 (0.13)
nSEP, mean (SD)	0.28 (0.82)	61.33 (5.73)

a Defined as consumption of fruits and vegetables every day (SAPALDIA) and consumption of at least 400 g of fruits and 400 g of vegetables per day (HAPIEE).

HAPIEE, Health, Alcohol and Psychosocial Factors in Eastern Europe; NDVI, normalized difference vegetation index; nSEP, neighborhood socioeconomic position; SAPALDIA, Swiss cohort Study on Air Pollution and Lung and Heart Diseases in Adults; WHO, World Health Organization.

### Prevalence of MetS in Czech and Swiss study populations

Prevalence of MetS was substantially higher in Czech participants (51.1%) compared with Swiss participants (35.8%, Table 2). Except for elevated TG, all metabolic components were more prevalent in HAPIEE than in SAPALDIA. Abdominal obesity and hypertension were the most prevalent components in both cohorts (Table 2). We observed elevated blood pressure in 37.8% HAPIEE and 19.7% SAPALDIA participants despite being previously diagnosed or treated for hypertension. While persons with high blood pressure in the absence of a hypertension diagnosis/treatment was equally high in both cohorts (over 30%), a substantially higher proportion of underdiagnosed diabetes cases was observed in the HAPIEE compared with the SAPALDIA cohort (20.0% vs. 4.2%). Correlation coefficient between the metabolic components ranged from 0.176 to 0.671 (*P* < 0.001) in HAPIEE and 0.217 to 0.690 (*P* < 0.001) in SAPALDIA (Tables S3 and S4; https://links.lww.com/EE/A343).

**Table 2.** Prevalence of metabolic syndrome and the components (%)

	HAPIEE	SAPALDIA
	N = 4931	N = 4442
Metabolic syndrome, %		
Yes	51.1	35.8
No	48.9	64.2
Abdominal obesity, %		
Yes	70.9	61.1
No	29.1	38.9
Hypertension, %		
Yes	78.6	58.9
No	21.4	41.1
Diagnosed/treated and BP <130/85 mmHg	6.1	7.1
Diagnosed/treated and BP ≥130/85 mmHg	37.8	19.7
Not diagnosed/treated and BP <130/85 mmHg	21.4	41.1
Not diagnosed/treated and BP ≥130/85 mmHg	34.7	32.1
Diabetes, %		
Yes	31.1	9.4
No	68.9	90.6
Diagnosed and glycemia <5.6 mmol/L	4.7	4.0
Diagnosed and glycemia ≥5.6 mmol/L	6.4	1.2
Not diagnosed and glycemia <5.6 mmol/L	68.9	90.6
Not diagnosed and glycemia ≥5.6 mmol/L	20.0	4.2
Impaired HDL, %		
Yes	34.5	28.3
No	65.5	71.7
Treated and HDL ≥1.03 in men and ≥1.29 in women	8.4	8.2
Treated and HDL <1.03 in men and <1.29 in women	4.2	2.8
Not treated and HDL ≥1.03 in men and ≥1.29 in women	65.5	71.7
Not treated and HDL <1.03 in men and <1.29 in women	21.9	17.3
Elevated TG, %		
Yes	48.4	51.1
No	51.6	48.9
Treated and TG <1.7 mmol/L	5.8	3.7
Treated and TG ≥1.7 mmol/L	6.8	7.3
Not treated and TG <1.7 mmol/L	51.6	48.9
Not treated and TG ≥1.7 mmol/L	35.8	40.1

Metabolic syndrome defined as abdominal obesity and any 2 phenotypes.

Abdominal obesity defined as waist ≥ 94 cm in men and ≥ 80 cm in women.

BP, blood pressure; HAPIEE, Health, Alcohol and Psychosocial Factors in Eastern Europe; HDL, high-density lipoprotein; SAPALDIA, Swiss cohort Study on Air Pollution and Lung and Heart Diseases in Adults; TG, triglycerides.

### Associations of environmental exposures with MetS and its components

**Figure 2.**
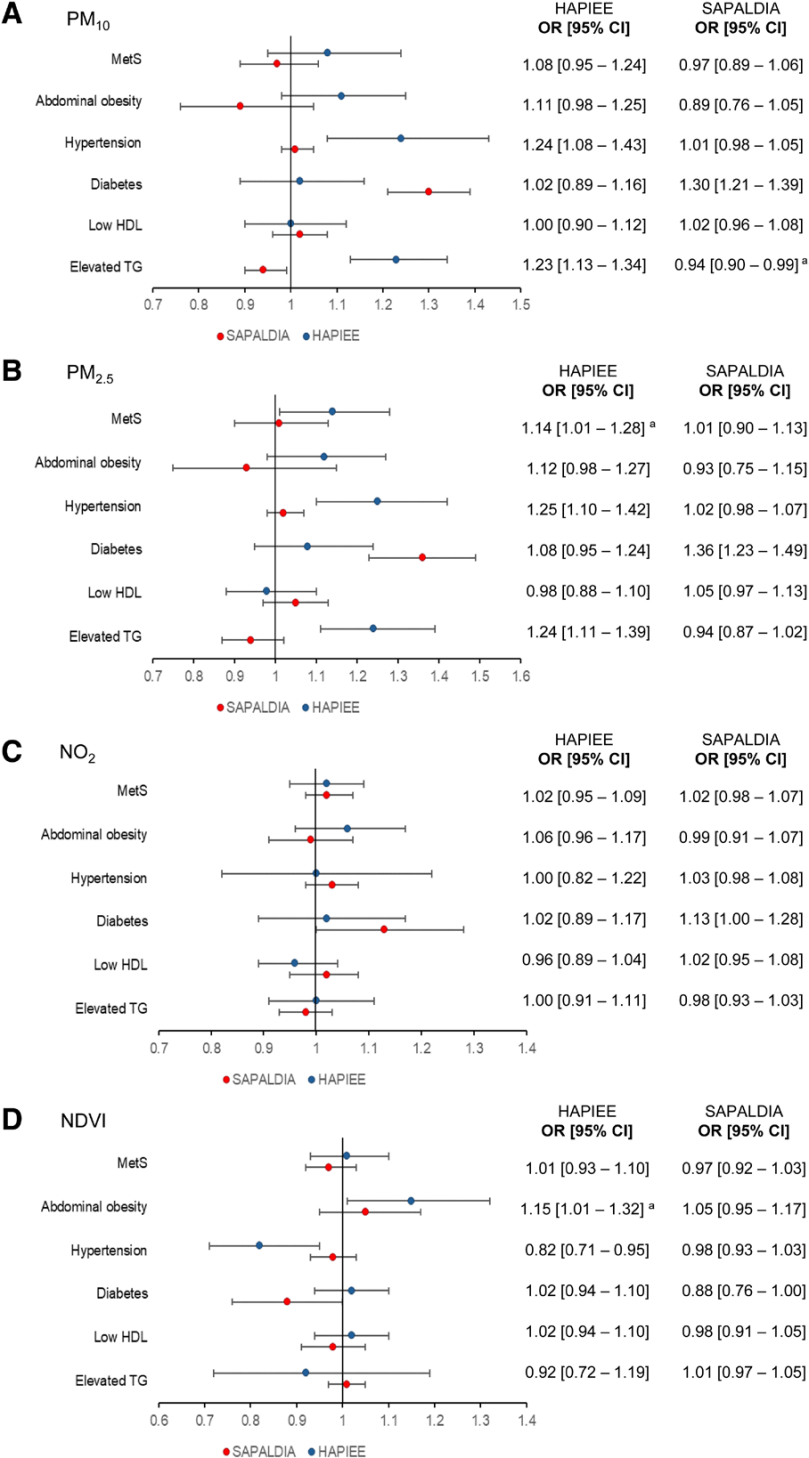
Logistic regression results adjusted for sex, age, education, marital status, smoking status, physical activity, alcohol consumption, fruit and vegetable consumption, nSEP, and month of the year. OR and 95% CI represent an increase in odds of outcomes per 5 μg/m^3^ of PM_10_, PM_2.5_, NO_2,_ and 0.1 NDVI. ^a^ Lost statistical significance following Bonferroni correction at *P* < 0.008 (0.05/6). CI, confidence interval; NDVI, normalized difference vegetation index; nSEP, neighborhood socioeconomic position; OR, odds ratio.

Figure 2 summarizes the fully adjusted OR and 95% CI for MetS and its components for PM_10_, PM_2.5_, NO_2_, and NDVI. In HAPIEE, a 5 μg/m^3^ increase in PM_2.5_ was associated with 14% higher odds of MetS (OR = 1.14; 95% CI = 1.01, 1.28). No significant associations were observed for PM_10_ and NO_2_ exposures. In SAPALDIA, evidence of null associations between air pollutants and increased odds of MetS was found (e.g. OR = 1.01; 95% CI = 0.90, 1.13 for PM_2.5_). Finally, null associations were found between NDVI and MetS in any of the studies (e.g. for SAPALDIA: OR = 0.97; 95% CI = 0.92, 1.03). For the association of other covariates with MetS, see Table S5; https://links.lww.com/EE/A343.

Among the MetS components, we observed mixed findings between cohorts (Figure 2). In HAPIEE, 5 μg/m^3^ of PM_10_ and PM_2.5_ was associated with 24% (OR = 1.24; 95% CI = 1.08, 1.43) and 25% (OR = 1.25; 95% CI = 1.10, 1.42) increased odds of hypertension, respectively. In contrast, we found no association of PM_10_ and PM_2.5_ exposures with hypertension in SAPALDIA. Additionally, null associations were observed between air pollutants and diabetes in HAPIEE, while 5 μg/m^3^ of PM_10_, PM_2.5_, and NO_2_ were associated with 30% (OR = 1.30; 95% CI = 1.21, 1.39), 36% (OR = 1.36; 95% CI = 1.23, 1.49), and 13% (OR = 1.13; 95% CI = 1.00, 1.28) increased odds of diabetes in SAPALDIA, respectively. In HAPIEE, 5 μg/m^3^ of PM_10_ and PM_2.5_ were associated with 23% (e.g., OR = 1.23; 95% CI = 1.13, 1.34) and 24% (e.g., OR = 1.24; 95% CI = 1.11, 1.39) increased odds of elevated TG, respectively. In contrast, a negative association was observed between PM_10_ and elevated TG in SAPALDIA (OR = 0.94; 95% CI = 0.90, 0.99). No associations were observed between NO_2_ exposure and the MetS components in any of the cohorts, except for the positive association with diabetes in SAPALDIA. Additionally, we found contradictory findings on NDVI exposure and its association with MetS components. In HAPIEE, the odds of hypertension decreased by 18% (OR = 0.82; 95% CI = 0.71, 0.95), while the odds of abdominal obesity increased by 15% (OR = 1.15; 95% CI = 1.01, 1.32) per 0.1 NDVI. In SAPALDIA, null associations were identified for the relationship between greenness exposure and the MetS components, except for the inverse association with diabetes (OR = 0.88; 95% CI = 0.76, 1.00) (Figure 2).

### Sensitivity analysis

The Poisson regression revealed comparable findings on the associations of air pollutants and greenspace exposure with MetS (Table S6; https://links.lww.com/EE/A343). Excluding participants who had previously been diagnosed or treated for MetS components did not noticeably change the associations between exposures and outcomes (Table S7; https://links.lww.com/EE/A343).

## Discussion

### Summary

Despite the higher prevalence of several MetS risk factors in SAPALDIA, we observed a substantially higher prevalence of MetS in Czech individuals, who were additionally more likely to be underdiagnosed and uncontrolled for hypertension and diabetes. PM_2.5_ exposure was substantially higher and positively associated with MetS in HAPIEE, while no association was observed in SAPALDIA. Moreover, in any of the studies, we did not find evidence for associations between exposures to greenspace, PM_10_, NO_2,_ and MetS. Importantly, we observed mixed findings between the cohorts in the associations of air pollutants and greenness on MetS components. We observed no evidence about associations between NO_2_ exposure and MetS components in HAPIEE and only a borderline association between NO_2_ and diabetes in SAPALDIA. Finally, increased exposure to greenspace was associated with decreased risk of hypertension in HAPIEE, and diabetes in SAPALDIA.

### Comparisons of findings between study cohorts

The observed heterogeneity in associations between air pollution and greenspace with MetS and its components is in line with the inconsistency in findings in previous studies (see *Comparisons with previous literature*). Given the comparable number of participants, similar age distribution of the study samples, consistent time points of the participants’ enrollments, and harmonized exposure estimation methods, these factors are not likely to explain the observed differences.

Other explanations may underlie the reported differences in the associations observed for MetS and its components. First, the composition of PM may play a substantial role in the adverse effects on MetS. PM is a complex mixture of several components, including black carbon, nitrate, ammonium, sulfate, organic matter, and mineral dust that may impact MetS differently. Previous studies on Chinese adults identified black carbon as the most detrimental component of PM.^41,42^ As dominant anthropogenic sources of PM are expected to vary between Western and Eastern European countries, with the greatest contribution of transport emissions in Switzerland and residential heating and agriculture in the Czech Republic,^43^ the proportion of the most harmful components may play a substantial role in explaining the differences in the associations we observed. The oxidative potential and subsequent physiological responses of particulate matter may substantially differ depending on its composition. Understanding these differences is crucial for developing context-specific public health strategies aimed at reducing exposure and mitigating health risks associated with air pollution exposure.^44,45^ Second, we observed a substantially higher proportion of Czech compared with Swiss participants who were poorly controlled or underdiagnosed for hypertension. These individuals are more likely to experience concomitant symptoms that may intensify susceptibility to harmful effects of air pollution exposures.^44,46^ Additionally, these individuals may be less likely to utilize greenspaces for leisure and physical activities. Yet, the restriction of the analyses to untreated and undiagnosed participants did not materially alter the main findings reported.^45^ Third, the various findings observed for greenspace among cohorts may reflect differences in the characterization of NDVI that may capture various types of greens in the studied areas.^47,48^ Finally, intra- and inter-population socioeconomic inequalities are well-documented stressors increasing the risk of cardiometabolic diseases. While we adjusted our analysis for education, residual confounding may be present by ignoring socioeconomic deprivation and occupation position, known predictors of psychological distress, and unhealthy behaviors contributing to a higher prevalence of noncommunicable diseases. If residual confounding differed between the two cohorts, or if economic, environmental, behavioral, and health contexts modify the effects of air pollution and greenspace differentially between cohorts, the average environmental exposure-MetS associations would also vary.

### Comparisons with previous literature

Inconsistent findings of the associations between air pollution exposures and MetS have been shown previously.^9^ The results of Heinz Nixdorf Recall Study^17^ and 33 Communities Chinese Health Study ^49^ identified positive, but weak associations between PM_2.5_ and MetS (ORs = 1.14; 95% CI = 0.99, 1.32, and ORs = 1.09; 95% CI = 1.00, 1.18), respectively. On the contrary, KORA (Cooperative Health Research in the Region Augsburg) F4/FF4 study reported positive associations of PM_2.5_ with MetS (OR = 1.14; 95% CI = 1.02, 1.28).^18^ In the SAPALDIA cohort, we previously reported 31% and 17% increased odds of MetS per 10 μg/m^3^ increase in PM_10_ and NO_2_, respectively.^19^ The inconsistent findings with our current study may be explained by applying different exposure estimation methods, exposure duration, and time window. In particular, the previous study utilized dispersion models for estimating 10-year average concentrations (1990–2000) of the air pollutants, whereas the current study used data from land-use regression models and applied 1-year average concentrations before the examination (2000–2005). Additionally, the inconsistency may be explained by applying a different set of selected confounders, most importantly adjustment for other inhalants, including exposure to environmental tobacco smoke and occupational gas, dust, or fumes exposure.

Although there is no consensus established for the MetS-air pollution association, several systematic reviews and meta-analyses provided evidence for the relationships between long-term air pollution exposure and the individual metabolic components. An umbrella review of seven systematic reviews and meta-analyses indicated a positive association between air pollution exposure and weight status (obesity and overweight).^50^ Likewise, recent meta-analyses pooled data from 57 epidemiological studies. They reported an increased risk of hypertension associated with increased exposure to PM_2.5_, and PM_10_, but not to NO_2_, NO_x,_ and PM_2.5–10_ which is aligned with our observations.^51^ Additionally, another meta-analysis of 21 eligible cohort studies indicated higher diabetes risks linked with elevated exposure to NO_2_, while inconclusive findings were reported for PM_10_ and PM_2.5_ aligned with the observation in the SAPALDIA sample.^52^ Evidence about associations between exposure to air pollution and altered levels of TG and HDL cholesterol remains scarce. The meta-analyses including results from three studies revealed increased PM_10_ and NO_2_ exposure to be associated with elevated TG levels, while no associations were detected between air pollutants and impaired HDL cholesterol.^53^ These findings are consistent with our results observed in the cohorts.

So far, epidemiological studies investigating the association between greenspace and MetS are scarce, and the findings are inconsistent.^14^ The results of several Chinese studies and one UK study investigating middle-aged participants revealed a negative association between greenness exposure and risk of MetS.^22,54–56^ In contrast, a study investigating German participants reported no significant association,^18^ which is aligned with our results observed in the Czech and Swiss populations. The inconsistency of the previous findings may be potentially explained by inappropriate adjustment for behavioral variables and area-level SEP when estimating the effect of a greenspace exposure to MetS. Furthermore, none of the studies, including ours, reported accessibility and utilization of greenspaces that might over- or underestimate the effect on MetS due to effect modification.^14^ None of the studies characterized the type of greenspace in more detail, which plays an important role in its access and utilization.^14^

The relationships between long-term exposure to greenspaces and the individual metabolic components have been reported previously. The results of a recent meta-analysis of 23 studies using NDVI showed beneficial associations of increased NDVI on blood pressure and hypertension,^57^ consistent with our findings of the HAPIEE cohort, but not SAPALDIA. Access to greenspace is generally associated with lower BMI and reduced risk of obesity.^58^ Our results showed, however, a null and the opposite associations in SAPALDIA and HAPIEE, respectively. These mixed findings were reported previously, suggesting a significant effect modification by gender; greenness was associated with a reduced likelihood of obesity among women with an increased likelihood of obesity in men.^59^ Furthermore, the pooled results of three cross-sectional and one longitudinal study showed protective associations with diabetes,^60^ which is consistent with our findings on SAPALDIA. The authors of the reviews generally detected high between-study heterogeneity, thus reducing the credibility of the pooled evidence, coinciding with the heterogeneity of associations observed between HAPPIEE and SAPALDIA. Finally, evidence of the association between greenspace exposure and dyslipidemia and impaired HDL remains limited. A study conducted on Australian adults did not reveal a relationship between greenness and dyslipidemia,^61^ while the recent study investigating UK adults observed a negative association between greenspace exposure and both, high triglyceride levels and impaired HDL cholesterol.^56^

### Biological mechanisms

Experimental studies have proposed several possible biological mechanisms linking air pollution to MetS. Fine particles and soluble constituents can pass through the alveolar membrane into the circulatory system, directly enhancing systemic inflammation and oxidative stress.^11,12^ The inflammation responses may result in elevated blood pressure, endothelial dysfunction, insulin resistance, and disorder of lipid metabolism, leading to dyslipidemia, diabetes, and obesity.^51,53^ Importantly, a previous study has suggested that individuals with MetS have even greater susceptibility to air pollution, possibly increasing cardiovascular risk.^12^ In particular, the study conducted on the US population found that participants with MetS showed stronger positive C-reactive protein responses than those without MetS.^12^ Further, laboratory research has shown that air pollutants, particularly PM, provoke oxidative stress; a well-established causative factor of numerous associated adverse health outcomes, including MetS. It has been suggested that oxidative stress is triggered by mitochondria as a primary target of particulate matter and consequently exacerbates DNA damage and other mutagenic activities.^62^ Moreover, exposure to air pollution can affect the autonomic nervous system, leading to alterations in heart rate variability and blood pressure regulation.^63^

Several underlying mechanisms have been proposed for the association between greenspace exposure and MetS. Greenspace is thought to prevent MetS by promoting physical activity,^15^ a well-established protective factor of obesity, hypertension, dyslipidemia, and type 2 diabetes.^64^ In addition, previous studies suggested that greenspace may mitigate exposure to air pollutants and noise.^65^ Furthermore, exposure to greenspace might reduce chronic stress,^15^ contributing to decreased risk of MetS.^66^ Finally, exposure to greenspace has been proposed to enhance social engagement,^67^ previously linked with decreased risk of MetS.^68^

### Strengths and limitations of the study

The HAPIEE and SAPALDIA studies are well-characterized, population-based cohorts offering extensive social and lifestyle data databases, enabling us to adjust for multiple potential confounders. To the best of our knowledge, this is the first study that conducted a comparative analysis of Western and Eastern European countries, providing evidence of relationships between environmental characteristics and prevalence of MetS using harmonized exposure estimation methods and a unified MetS definition. In addition, we conducted a sensitivity analysis on samples of participants who were not previously diagnosed or treated for the respective MetS components. This sensitivity analysis confirmed the robustness of our findings.

Several limitations need to be acknowledged. First, a major limitation of our study is the cross-sectional design that did not allow us to examine the causal associations or their temporality. Second, there is no consensus on the exact MetS definition leading to a possible misclassification of the outcome. Previous studies reported different results depending on the MetS definition, with the WHO definition having the highest magnitude of the effects between the exposure and outcome.^19^ Given the fact that the WHO definition emphasizes insulin resistance as the major underlying risk factor, and given that the prediction model was applied in HAPIEE to estimate fasting venous glycemia using glucose levels from capillary blood, it is likely that applying WHO criteria would introduce a bias.^19^ The ATP III definition uses less strict criteria of abdominal obesity with cutoffs ≥102 cm in men and ≥88 cm in women, which would overestimate MetS prevalences in both cohorts.^27^ Moreover, waist circumference was not measured at the examination time in SAPALDIA; instead, it was estimated using a validated prediction model using trends from the next follow-up visit. Third, we did not have estimates of indoor or occupational air pollution for participants in both studies, which may be considered as a potential source of misclassification of air pollution exposure. To reduce the risk of bias related to occupational exposures, we excluded participants from heavily polluted industrial areas of the Czech Republic with a long history known for mining activities and heavy industry with potentially different susceptibility profiles and potentially different air pollution composition. This may limit the generalizability of the observed associations to the broader Czech population. When we included these regions in sensitivity analysis, the associations of PM_10_ and PM_2.5_ with diabetes also became positive in the HAPPIE cohort (data not shown). Fourth, no information regarding mobility patterns of the individuals was available. Mobility may substantially affect the cumulative exposures to indoor/outdoor air pollutants as well as exposure to greenspace. Additionally, our study assessed only some aspects of greenspace exposure, ignoring essential characteristics like type of greenspace and related accessibility and perceived utilization. Fifth, the environmental exposures considered in the analysis were limited to air pollution and greenspace. Unfortunately, information on transportation noise was not available in a harmonized manner for both cohorts. Both air pollution and transportation noise have been associated with cardiovascular phenotypes, possibly because of transportation-derived air pollution. However, the pattern of associations (and potential confounding) may differ between the two cohorts, as these exposures share pathological effect mechanisms with MetS.^69,70^ Yet, according to a recent systematic review, noise and air pollution may have interactive effects, which may explain some of the heterogeneity observed in the associations between the two cohorts.^71^ Sixth, there are additional limitations related to the selected confounders. Only frequency of alcohol consumption was considered, ignoring the actual alcohol content in the beverages and possible binge alcohol drinking. No detailed nutritional information, including consumption of processed food, snacks, etc., was available in the studies. Hidden healthy and social behavior factors might explain the differences in the prevalence of MetS between the two cohorts. Finally, as in any cohort selection bias in the cohort at study entry may limit the generalizability of the findings. Attrition bias due to death or nonparticipation at the first follow-up in SAPALDIA2 may have biased the results in the sense that participants at follow-up were survivors. Older, better-educated participants, nonsmokers, except for higher levels of BMI, and individuals less exposed to air pollutants at study entry were more likely to participate at the first follow-up (SAPALDIA2), confirming the tendency for follow-up participants to be slightly healthier (Table S8; https://links.lww.com/EE/A343). Similar findings were found in the HAPIEE study, although the included and excluded participants were of the same age, and a higher proportion of individuals with normal BMI was included in the analysis (Table S8; https://links.lww.com/EE/A343). However, attrition bias may have contributed to an underestimation of certain effects in both studies and to the observed heterogeneity of effects between the two cohorts.^19,26^

## Conclusion

A much higher prevalence of MetS as well as of underdiagnosed and uncontrolled hypertension and diabetes was observed in HAPIEE (Czech Republic) compared with SAPALDIA (Switzerland). HAPIEE participants were exposed to higher particulate matter exposure with potentially different sources and compositions. A positive association between PM_2.5_ and MetS was observed in the Czech Republic only, but not in Switzerland. The differential associations of air pollution with MetS components between the two studies may reflect air pollution source and composition differences. Future studies are encouraged to utilize the existing harmonized exposure datasets developed within the EXPANSE project. Additionally, greater attention should be paid to pollution sources and the composition of both, air pollution and greenspace. The study confirms the value of including cohorts from different economic, environmental, behavioral, and health systems contexts for adapting environmental impact assessment.

## Conflicts of interest statement

The authors declare that they have no conflicts of interest with regard to the content of this report.

## Supplementary Material
