# Supplementary material for: Air pollution, greenspace, and metabolic syndrome in older Czech and Swiss populations
Source: Environ Epidemiol. 2025 May 8;9(3):e393. doi: 10.1097/EE9.0000000000000393 (PMC12063789; doi:10.1097/EE9.0000000000000393)
Supplement: Supplementary file 1 [file ee9-9-e393-s001.pdf]

Air pollution, greenspace, and metabolic syndrome in older Czech and Swiss populations

Supplementary material

Table S1. Spearman correlation coefficients between environmental exposures (HAPIEE, N=4,931)

	PM_{10}	$PM_{2.5}$	NO_2	NDVI	nSEP
PM_{10}	1				
$PM_{2.5}$	0.848*	1			
NO_2	0.028	-0.095*	1		
NDVI	-0.424*	-0.347*	-0.208*	1	
nSEP	0.168*	0.251*	0.008	-0.125*	1
* p-value < 0.001					

Table S2. Spearmen correlation coefficients between environmental exposures (SAPALDIA, N=4,442)

	PM_{10}	$PM_{2.5}$	NO_2	NDVI	nSEP
PM_{10}	1				
$PM_{2.5}$	0.899*	1			
NO_2	0.783*	0.826*	1		
NDVI	-0.630*	-0.702*	-0.731*	1	
nSEP	0.142*	0.192*	0.385*	-0.004	1
* p-value < 0.001					

Table S3. Tetrachoric correlation coefficients between components of the metabolic syndrome (HAPIEE, N=4,931)

	METS	Abdominal obesity	Hypertension	Diabetes	Low HDL	Elevated TG
METS	1					
Abdominal obesity	0.864*	1				
Hypertension	0.576*	0.386*	1			
Diabetes	0.580*	0.214*	0.275*	1		
Low HDL	0.626*	0.364*	0.211*	0.183*	1	
Elevated TG	0.705*	0.394*	0.357*	0.176*	0.671*	1
* p-value < 0.001						

Table S4. Tetrachoric correlation coefficients between components of the metabolic syndrome (SAPALDIA, N=4,442)

	METS	Abdominal obesity	Hypertension	Diabetes	Low HDL	Elevated TG
METS	1					
Abdominal obesity	1.000*	1				
Hypertension	0.708*	0.434*	1			
Diabetes	0.554*	0.288*	0.354*	1		
Low HDL	0.734*	0.366*	0.300*	0.249*	1	
Elevated TG	0.849*	0.404*	0.374*	0.217*	0.690*	1
* p-value < 0.001						

Table S5. The associations between covariates and metabolic syndrome

	HAPIEE N=4,931		SAPALDIA N=4,442	
	OR (95% CI)	p-value	OR (95% CI)	p-value
**Age**	1.06 (1.06 – 1.07)	<0.001	1.07 (1.06 – 1.08)	<0.001
**Gender** (ref. Male)				
Female	0.77 (0.68 – 0.87)	<0.001	0.50 (0.42 – 0.60)	<0.001
**Education** (ref. Incomplete primary/primary)				
Vocational/secondary	0.76 (0.62 – 0.92)	0.005	0.75 (0.57 – 0.99)	0.044
University or higher	0.49 (0.38 – 0.65)	<0.001	0.50 (0.41 – 0.63)	<0.001
**Marital status** (ref. Married or cohabiting)				
Divorced/separated	0.80 (0.64 – 1.01)	0.056	1.11 (0.85 – 1.44)	0.438
Widowed	1.02 (0.77 – 1.33)	0.908	1.01 (0.81 – 1.25)	0.957
Single	0.78 (0.53 – 1.17)	0.222	0.77 (0.61 – 0.96)	0.022
**Smoking status** (ref. never)				
Former	1.33 (1.21 – 1.46)	<0.001	1.32 (1.12 – 1.56)	0.001
Current or occasional	1.13 (0.94 – 1.345	0.191	1.26 (1.06 – 1.51)	0.009
**Physical activity** (ref. Inactive)				
Active	0.73 (0.62 – 0.86)	<0.001	0.84 (0.74 – 0.95)	0.005
**Alcohol frequency** (ref. < Once per month)				
≥ Once per month	0.89 (0.84 – 0.95)	<0.001	0.77 (0.63 – 0.93)	0.007
**Fruit and vegetable consumption (ref. Low consumption)**				
Normal consumption	1.17 (1.10 – 1.56)	<0.001	1.18 (1.07 – 1.30)	0.001
**nSEP**	0.93 (0.90 – 0.96)	<0.001	0.98 (0.97 – 0.99)	<0.001
Model adjusted for sex, age, education, marital status, smoking status, physical activity, alcohol consumption, fruit and vegetable consumption, nSEP, month of the year, PM_{10}, $PM_{2.5}$, NO_2, and NDVI. nSEP: Neighborhood-level socio-economic position				

Table S6. The associations between environmental exposures and metabolic syndrome using Poisson regression

	HAPIEE N=4,931		SAPALDIA N=4,442	
	IRR (95% CI)	p-value	IRR (95% CI)	p-value
PM_{10}	1.04 (0.98 – 1.11)	0.233	0.98 (0.94 – 1.03)	0.464
$PM_{2.5}$	1.06 (1.00 – 1.12)	0.038	1.00 (0.94 – 1.07)	0.894
NO_2	1.01 (0.98 – 1.04)	0.629	1.01 (0.99 – 1.04)	0.372
NDVI	1.00 (0.97 – 1.04)	0.842	0.99 (0.95 – 1.02)	0.388

Poisson regression with cluster robust SE
Model adjusted for sex, age, education, marital status, smoking status, physical activity, alcohol consumption, fruit and vegetable consumption, nSED, and month of the year.
Note: IRR values represent fold increase in odds of outcomes per 5 $\mu g/m^3$ of PM_{10}, $PM_{2.5}$, NO_2, and 0.1 NDVI.

Table S7. The associations between environmental exposures and components of metabolic syndrome, diagnosed and/or treated participants excluded

		**Abdominal obesity**		**Hypertension**		**Diabetes**		**Low HDL**		**Elevated TG**	
	Cohort	OR (95% CI)	p-value	OR (95% CI)	p-value	OR (95% CI)	p-value	OR (95% CI)	p-value	OR (95% CI)	p-value
PM_{10}	HAPIEE	1.05 (0.87 – 1.27)	0.602	1.24 (1.10 – 1.40)	0.001	0.93 (0.83 – 1.04)	0.197	0.95 (0.87 – 1.04)	0.283	1.09 (0.96 – 1.24)	0.172
	SAPALDIA	0.89 (0.77 – 1.02)	0.083	0.99 (0.91 – 1.08)	0.874	1.28 (1.00 – 1.64)	0.047 [a]	0.96 (0.89 – 1.04)	0.393	0.95 (0.90 – 1.00)	0.047 [a]
$PM_{2.5}$	HAPIEE	1.03 (0.83 – 1.28)	0.795	1.23 (1.09 – 1.39)	0.001	0.91 (0.77 – 1.08)	0.291	0.97 (0.87 – 1.08)	0.545	1.13 (1.00 – 1.27)	0.043 [a]
	SAPALDIA	0.91 (0.75 – 1.10)	0.301	0.97 (0.88 – 1.08)	0.608	1.34 (0.93 – 1.93)	0.113	0.96 (0.87 – 1.07)	0.468	0.93 (0.89 – 0.98)	0.003
NO_2	HAPIEE	1.11 (0.99 – 1.25)	0.067	1.04 (0.84 – 1.29)	0.694	1.06 (0.96 – 1.72)	0.254	0.97 (0.88 – 1.07)	0.572	1.00 (0.92 – 1.09)	0.971
	SAPALDIA	0.97 (0.90 – 1.05)	0.496	1.02 (0.91 – 1.13)	0.770	1.12 (0.95 – 1.31)	0.178	0.97 (0.89 – 1.06)	0.526	0.97 (0.92 – 1.02)	0.221
NDVI	HAPIEE	1.22 (1.07 – 1.39)	0.003	0.77 (0.69 – 0.85)	<0.001	1.12 (1.02 – 1.23)	0.023 [a]	1.05 (0.88 – 1.24)	0.587	0.98 (0.71 – 1.34)	0.895
	SAPALDIA	1.07 (0.96 – 1.18)	0.216	0.98 (0.90 – 1.06)	0.610	0.87 (0.71 – 1.06)	0.154	0.99 (0.92 – 1.07)	0.822	1.02 (0.97 – 1.06)	0.500

Multivariable logistic regression with cluster-robust SE.
HDL: high-density lipoprotein cholesterol; TG: triglycerides
Model included participants who were not diagnosed or treated for none of the listed components (hypertension, diabetes, low HDL cholesterol or elevated triglycerides). The components are defined based on measured biomarkers only (N=2,398 for HAPIEE and N=3,066 for SAPALDIA).
Model adjusted for sex, age, education, marital status, smoking status, physical activity, alcohol consumption, fruit and vegetable consumption, nSED and month of the year.
Note: OR values represent fold increase in odds of outcomes per 5 $\mu g/m^3$ of PM_{10}, $PM_{2.5}$, NO_2, and 0.1 NDVI.
[a] Lost statistical significance following Bonferroni correction at $p<0.008$ (0.05/6).

Table S8: Characteristics of participants included and excluded in the HAPIEE and SAPALDIA studies

	HAPIEE			SAPALDIA		
	**Included** N=4,931	**Excluded** N=3,925	**P-value** (Chi^2 or t-test)	**Included** N=4,442	**Excluded** N=5,209	**P-value** (Chi^2 or t-test)
**Age**, mean (SD)	58.19 (7.15)	58.19 (7.16)	0.98	46.6 (7.8)	36.3 (12.3)	<0.001
**Gender, %**						
Females	54.8	51.7		51.8	50.0	
Males	45.2	48.3	0.004	48.2	50.0	0.08
**BMI category**, %						
Underweight (<18.5)	0.2	0.3		2.5	4.5	
Normal (18.5-25)	24.4	21.3		60.2	64.6	
Overweight (25-30)	44.8	46.5		30.5	23.8	
Obese (≥30)	30.6	31.9	0.07	6.8	7.2	<0.001
**Education**, %						
Primary	9.9	16.5		15.5	18.1	
Secondary	74.4	72.6		66.3	66.5	
University	15.7	10.9	<0.001	18.2	15.4	<0.001
**Marital status**, %						
Married/Cohabiting	75.8	75.2		78.3	51.2	
Divorced/Separated	12.6	12.5		7.4	5.8	
Widowed	8.9	9.9		2.2	1.4	
Single	2.7	2.4	0.467	12.1	41.6	<0.001
**Smoking status**, %						
Never	45.2	42.4		43.9	43.9	
Former	30.1	28.7		27.2	18.7	
Current/Occasional	24.7	28.9	<0.001	28.9	37.5	<0.001
**Air pollutants** [μg/m^3], mean (SD)						
PM_{10}	35.94 (4.08)	42.14 (9.81)	<0.001	43.8 (9.67)	44.7 (8.93)	<0.001

$PM_{2.5}$	26.62 (3.91)	33.40 (9.67)	<0.001	30.0 (8.91)	31.0 (8.11)	<0.001
NO_2	26.47 (4.44)	25.95 (4.21)	<0.001	34.1 (10.7)	36.7 (11.8)	<0.001
**Greenness,** mean (SD)						
NDVI, 500 m	0.45 (0.07)	0.47 (0.07)	<0.001	0.53 (0.14)	0.49 (0.14)	<0.001

S9 information: Additional acknowledgement

The HAPIEE cohort was established by the HAPIEE Team:

Study directorate: M Bobak (PI; e/s); H Pikhart (e/s)

Scientific team: A Peasey (e/s), R Kubinova (e), Sofia Mayutina (e), Andrzej Pajak (e), A Tamosiunas (e), A Nicholson (e), Michael Marmot (e)

Scientific team at coordinating centers: R Kubinova (e), N Capkova (e)

(a) allergology, (c) cardiology, (cc) clinical chemistry, (e) epidemiology, (exp) exposure, (g) genetic and molecular biology, (m) meteorology, (n) nutrition, (o) occupational health, (p) pneumology, (pa) physical activity, (pd) pediatrics, (s) statistics

The SAPALDIA cohort and biobank was established by the SAPALDIA Team:

Study directorate: NM Probst-Hensch (PI; e/g); D Stolz (p), C Schindler (s), N Künzli (e/exp)

Scientific team: JC Barthélémy (c), W Berger (g), R Bettschart (p), A Bircher (a), C Brombach (n), PO Bridevaux (p), L Burdet (p), Felber Dietrich D (e), T Sigrist (p), U Frey (pd), MW Gerbase (p), D Gold (e), E de Groot (c), W Karrer (p), F Kronenberg (g), B Martin (pa), A Mehta (e), D Miedinger (o), M Pons (p), F Roche (c), T Rothe (p), P Schmid-Grendelmeyer (a), A Schmidt-Trucksäss (pa), J Schwartz (e), A Turk (p), A von Eckardstein (cc), E Zemp Stutz (e).

Scientific team at coordinating centers: M Adam (e), I Aguilera (exp), A Beckmeyer-Borowko (e), S Brunner (s), D Carballo (c), S Caviezel (pa), I Curjuric (e), A Di Pascale (s), J Dratva (e), R Ducret (s), E Dupuis Lozeron (s), M Eeftens (exp), I Eze (e), E Fischer (g), M Foraster (e), M Germond (s), L Grize (s), S Hansen (e), A Hensel (s), M Imboden (g), A Ineichen (exp), A Jeong (g), D Keidel (s), A Kumar (g), N Maire (s), A Mehta (e), R Meier (exp), E Schaffner (s), T Schikowski (e), M Tsai (exp)

(a) allergology, (c) cardiology, (cc) clinical chemistry, (e) epidemiology, (exp) exposure, (g) genetic and molecular biology, (m) meteorology, (n) nutrition, (o) occupational health, (p) pneumology, (pa) physical activity, (pd) pediatrics, (s) statistics

Local fieldworkers S1 : Aarau: C Persoz-Borer, C Wettstein, G Giger, H Grob-Stalder, J Lohmüller, K Häfeli, U.Rippstein. Basel: V Fluri, M Herrous, G Imboden, L Joos. Davos: K D'Alberti, A Sönnichsen. Genf: I Barbey, K Gegere, N Penay. Lugano: M Astone, E Haechler, E Riesen, B Viscardi. Montana: C Hollenstein, E Borgeat, I Clivaz. Payerne: S Menétrey-Jaques, C Gilomen-Pages, MC Collaud. Wald: B Salzmann, V Kienast, H Astone, V Keller, C Schwalm.

Local fieldworkers S2 : Aarau: M Broglie, M Bünter, D Gashi. Basel: R Armbruster, T Damm, U Egermann, M Gut, L Maier, A Vögelin, L Walter, Davos: D Jud, N Lutz. Geneva: M Ares, M Bennour, B Galobardes, E Namer. Lugano: B Baumberger, S Boccia Soldati, E Gehrig-Van Essen, S Ronchetto, Montana: C Bonvin, C Burrus. Payerne: S Blanc, AV Ebinger, ML Fragnière, J Jordan. Wald: R Gimmi, N Kourkoulos, U Schafroth.

Local fieldworkers S3 : Aarau: S Brun, G Giger, M Sperisen, M Stahel. Basel: C Bürli, C Dahler, N Oertli, I Harreh, F Karrer, G Novicic, N Wyttenbacher. Davos: A Saner, P Senn, R Winzeler, Geneva: F Bonfils, B Blicharz, C Landolt, J Rochat. Lugano: S Boccia, E Gehrig, MT Mandia, G Solari, B

Viscardi. Montana: AP Bieri, C Darioly, M Maire., Payerne: F Ding, P Danieli A Vonnez. Wald: D Bodmer, E Hochstrasser, R Kunz, C Meier, J Rakic, U Schafroth, A Walder.

Local fieldworkers S4 : Aarau: A Heichel, E Isenring, C Marti, C Schuler, M Witzig. Basel: N Aebi, R Armbruster, S Nussbaumer, M Rutschi, M Witzig. Davos: S Derfler, M Dinic, L Esghani-Oswald, A Landauf. Geneva: N Ballot, B Ducry, M Plutino. Lugano: C Ferrari, I Paravac, P Parolini, T Vermes. Montana: P Friedrich, M Gérard, L Kristen, T Masserey, D Walther. Payerne: M Bertschy, C Bonzon, B Ducry, F Gainon, H Rodrigues. Wald: D Bodmer, C Kuntze, U Treder, A Lawson, S Mettler, D Studer, B Wigger.

Local fieldworkers S5 : Aarau, Basel, Davos: J. Brugger, C. Devonas, E. Kuper, N. Osswald, M. Pfeffermann, M. Ufer, L. Waschkowski, T. Wirth, M Witzig, T. Wuelser. Geneva, Montana, Payerne: N Ballot, M Gérard, M Plutino. Wald: U Treder, A Lawson.

Administrative staff: C Gabriel, R Gutknecht, N Bauer Ott,
